# Changes in the gut microbiome during fasting‐induced molting and its effects on gut–liver function

**DOI:** 10.1002/imo2.70103

**Published:** 2026-05-20

**Authors:** Hao Zhang, Zhixuan Luo, Jun Zhang, Chenxu Wang, Yadong Tian, Donghua Li, Yujie Guo, Yanhua Zhang, Wenting Li, Yujie Gong, Xiangtao Kang, Ruirui Jiang

**Affiliations:** ^1^ College of Animal Science and Technology Henan Agricultural University Zhengzhou China; ^2^ The Shennong Laboratory Zhengzhou China

**Keywords:** bile acid, fasting‐induced molting, gut microbiota, intestinal remodeling, liver function, metabolome, TGF‐beta signaling pathway

## Abstract

Fasting‐induced molting (FIM) rejuvenates the laying cycle in hens; however, the fasting process may result in intestinal dysbiosis, which compromises host immunity and disrupts the intestine‐liver function. Consequently, investigating the changes in gut microbiota during FIM and its impacts on gut‐liver metabolic homeostasis is crucial for enhancing the efficiency of FIM in laying hens. Here, a total of 90 laying hens, aged 60 weeks, were selected for the FIM. Samples were collected at four time points: the day before fasting, the 15th day of fasting, the 5th day of refeeding, and the 47th day of refeeding. Metagenomic sequencing and non‐targeted metabolomics were employed to investigate the roles of the gut microbiota and metabolites in the remodeling of gut–liver function, focusing on intestinal injury‐repair mechanisms and changes in liver function. During the fasting period, the abundance of harmful microbiota and metabolites increase, leading to intestinal injury. This process activates the TGF‐beta signaling pathway, promoting intestinal stem cell proliferation. Simultaneously, liver function dysfunction as evidenced by elevated bile acid levels in the liver and serum and activation of the non‐classical bile acid synthesis pathway. During the refeeding period, the previously observed effects were reversed, leading to the remodeling of the intestinal microbiota and gut‐liver function. We identified key microorganisms (*Liquorilactobacillus mali* and *Tissierellia* bacterium KA00581) and functional metabolites (d‐Panthenol and 3‐hydroxyanthranilic acid (3‐HAA)) involved in intestinal‐liver function during FIM. Notably, d‐Panthenol promotes chicken small intestinal organoid branching and growth, enhances barrier function, and reduces inflammation. Our study underscores the role of gut microbiota in gut‐liver injury during FIM in laying hens, suggesting potential probiotic‐ or metabolite‐based interventions to mitigate gut‐liver injury and facilitate recovery.

## Highlights

- This study establishes a dynamic profile of gut microbiota and metabolite changes during fasting‐induced molting (FIM).
- This study investigated the impact of the gut microbiota and metabolites on gut‐liver function during FIM.
- This study provides guidance for improving FIM management and feeding strategies to extend the lifespan of laying hens.

## Graphical abstract

During fasting, harmful microbiota and metabolites rise, triggering intestinal injury and activation of the TGF‐β signaling pathway, which enhances intestinal stem cell proliferation. Concurrently, hepatic function declines, indicated by increased hepatic and serum bile acids and upregulation of the non‐classical bile acid synthesis pathway. Upon refeeding, these perturbations reverse, driving remodeling of the gut microbiota and restoration of gut–liver homeostasis. This study investigated the impact of the gut microbiota and metabolites on gut–liver function during Fasting‐induced molting (FIM). The findings provide practical guidance for optimizing FIM management and feeding regimens to sustain laying‐hen health and productivity, with potential translational insights for gut–liver remodeling.

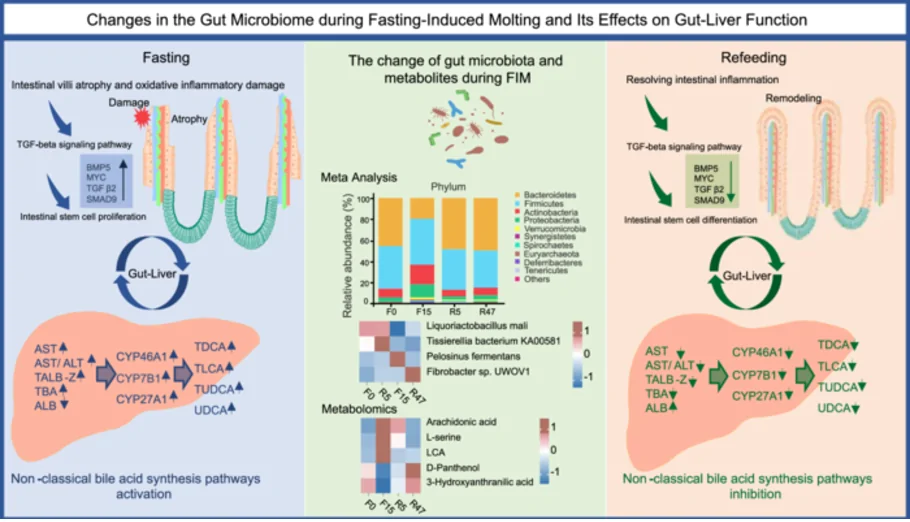

## 1 INTRODUCTION

Molting, a natural physiological phenomenon in poultry, involves feather replacement and a temporary cessation of reproduction, decreasing egg production [1]. Natural molting is typically prolonged and inconsistent, negatively affecting the maintenance of peak egg production and presenting challenges for intensive management [2]. Research has shown that penguins complete their feather molting by fasting for 2–5 weeks, maintaining optimal body conditions and feathers during breeding seasons to attract mates and protect offspring [3, 4]. Female poultry incubate their eggs without male assistance and are unable to leave the nest to forage, resulting in spontaneous anorexia [2]. During the incubation period, they enhance their resistance to hunger by reducing their activity and metabolic rates, indicating strong physiological and behavioral adaptations. Consequently, the induced molting process in chickens can be simulated by fasting to shorten the molting duration.

Fasting‐induced molting (FIM) is widely used in the egg industry because it offers a short cycle, an extended subsequent peak‐laying period, and reduced production costs. Additionally, FIM improves meat quality and slaughter traits of aged hens, thereby providing a basis for the sustainable utilization of aged laying hen resources [5]. However, accumulating evidence indicates that FIM can disrupt intestinal homeostasis and trigger gut microbiota dysbiosis [6, 7]. This dysbiosis involves increased pathogens, pro‐inflammatory metabolites, impaired barrier function, and mucosal inflammation, leading to microbial translocation and systemic inflammation [6, 8]. Importantly, gut microbiota regulates bile acid biotransformation and enterohepatic circulation; fasting‐induced dysbiosis may alter bile acid composition and signaling, thereby weakening hepatic immune homeostasis [9, 10]. This ultimately contributes to the dysfunction of the gut and liver, resulting in reduced immunity and higher mortality rates in chickens during FIM. Furthermore, the remodeling of gut‐liver function following refeeding is essential for laying hens to achieve a new peak‐laying period [11, 12, 13, 14]. Therefore, understanding how microbiota dysbiosis affects gut‐liver function is important for improving hens' health and performance during FIM.

To address these issues, we selected 90 aged laying hens for FIM. Metagenomic sequencing and non‐targeted metabolomics were used to analyze the changes of gut microbiota and metabolites. By integrating the mechanisms of intestinal injury‐repair and changes in liver function, we explored the roles of gut microbiota and metabolites in the gut–liver function remodeling process, identifying specific species and metabolites that influence FIM. Our findings elucidate a regulatory network governing gut microbiota's impact on gut‐liver health during FIM, providing a foundation for probiotic‐ or mrtabolite‐based interventions to mitigate damage and support remodeling during refeeding.

## 2 RESULTS

### 2.1 Gut microbial–metabolite dynamics during FIM

To investigate FIM‐induced gut microbiota alterations, we performed metagenomic sequencing of cecal contents. Fasting significantly altered cecal microbiota composition (Figure 1A–C), decreasing species diversity. At the phylum level, fasting reduced the abundance of *Bacteroidetes* but increased that of *Actinobacteria* and *Proteobacteria* (Figure 1D). At the genus level, the abundances of *Bacteroides* and *Prevotella* decreased, whereas those of *Olsenella*, and *Lachnoclostridium* increased during the fasting period (Figure 1E). Based on the top 50 microbial genera identified by the random forest model (Table S1), we observed stage‐specific dynamic correlations (Figure S1A). At F0, *Faecalimonas* was the dominant genus, showing a negative correlation with *Candidatus Woesearchaeota* noname. By F15, a shift occurred, with *Candidatus Aminicenantes* noname, *Enterobacter*, and *Slackia* rising to prominence; notably, *Slackia* displayed a negative correlation with *Arcanobacterium*. And, we identified the characteristic bacterial species by linear discriminant analysis effect size (LEfSe). For instance, *Escherichia coli*, *Lachnoclostridium* sp. An76, and *Cloacibacillus* sp. An23 were clustered during F15; *Lactobacillus crispatus, Ligilactobacillus salivarius, Ligilactobacillus aviarius*, and *Bacteroides uniformis* were clustered during R5 (LDA > 3 and *p* < 0.05) (Figure 1F). Next, Principal Co‐ordinates Analysis (PCoA) results showed that there was a difference in microbial function during FIM (Figure S1B). During fasting, pathways such as immune system and endocrine and metabolic disease were significantly enriched, indicating an increased susceptibility of the intestine to pathogenic invasion. During refeeding (R5 and R47), pathways related to replication and repair and lipid metabolism were significantly enriched, indicating improved adaptability of microorganisms in terms of energy metabolism and host health regulation (Figure S1C). These results indicate that pathogenic‐bacteria increase during fasting, and that probiotic levels rise again after refeeding.

**Figure 1:**
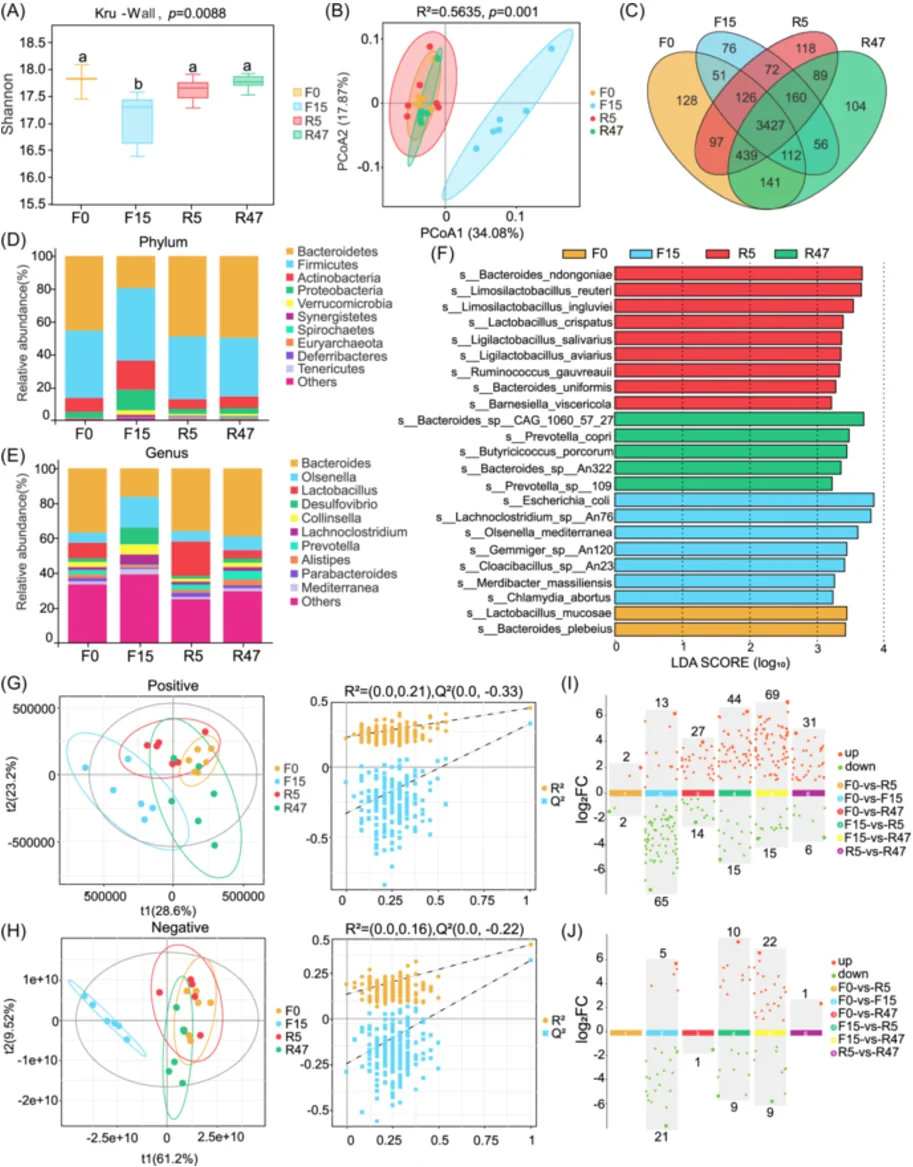
Alteration of the gut microbial and metabolites composition in laying hens during FIM. (A) Changes in alpha diversity during FIM. Alpha diversity of the microbiota was evaluated using the Shannon index by the Kruskal–Wallis test. (*p* < 0.05). F0, pre‐fasting; F15, Day 15 of fasting; R5, Day 5 of refeeding; R47, Day 47 of refeeding. (B) Principal coordinate analysis (PCoA) analysis based on unweighted UniFrac metrics of bacterial communities. (C) Difference Venn diagram of the relative comparison group. The relative abundance of cecal contents bacterial phylum (D), and genus (E). (F) Analysis of differences in species by Linear discriminant analysis effect size (LEfSe) in different periods (LDA > 3 and *q* < 0.05). (G, H) PLS‐DA analysis of cecal positive metabolites (G) and negative metabolites (H) based on Bray distance. Multiple scatter plot analysis was conducted to assess positive metabolites (I) and negative metabolites (J). *n* = 6.

Microorganisms are inextricably linked to metabolites, and the gut metabolites were significantly changed during FIM (Figure 1G–J). The enrichment of secondary bile acid biosynthesis during FIM points to a dysregulation of bile acid metabolism (Figure S1D). Based on the top 50 metabolites identified by the random forest model (Table S2), we observed stage‐specific dynamic correlations (Figure S1E). For instance, at F15, salicylic acid (SA) and N‐acetyl‐Asp‐Glu became prominent. N‐acetyl‐Asp‐Glu demonstrated a negative correlation with 4‐(1‐adamantyl)−2‐methyl‐1,3‐thiazol; at R47, 4‐(1‐adamantyl)−2‐methyl‐1,3‐thiazol became prominent. These findings reveal significant metabolic shifts and interrelationships across stages, highlighting the dynamic nature of the metabolic landscape in response to varying conditions.

### 2.2 Intestinal mucosa remodeling during FIM

Our findings demonstrate that fasting significantly impaired intestinal mucosa structure and function. Hematoxylin and eosin (H&E) staining revealed extensive atrophy and disruption in the jejunum and ileum during fasting (Figure 2A; Figure S2A), with Scanning electron microscopy (SEM) showing epithelial cell shedding (Figure 2B). Fasting led to reduced villus height and villus/crypt ratio, coupled with increased crypt depth (*p* < 0.05) (Figure 2C–E; Figure S2B–D). During refeeding, the jejunum and ileum gradually started remodeling from atrophy. We further investigated the inflammatory response and oxidative damage in the jejunum during FIM. During the fasting period, the mRNA expression levels of *P62*, *NF‐κB1*, *IL‐1β*, and *IL‐8*, as well as the protein level of IL‐1β, significantly increased (*p* < 0.05) (Figure 2F–K). Concurrently, the concentrations of catalase (CAT) and glutathione peroxidase (GSH‐PX) decreased (*p* < 0.05) (Figure 2L,M), while the concentration of malondialdehyde (MDA) increased (*p* < 0.05) (Figure 2N). Notably, the concentration of superoxide dismutase (SOD) remained unchanged during FIM (Figure 2O). These findings indicate that fasting induces intestinal inflammation and oxidative damage.

**Figure 2:**
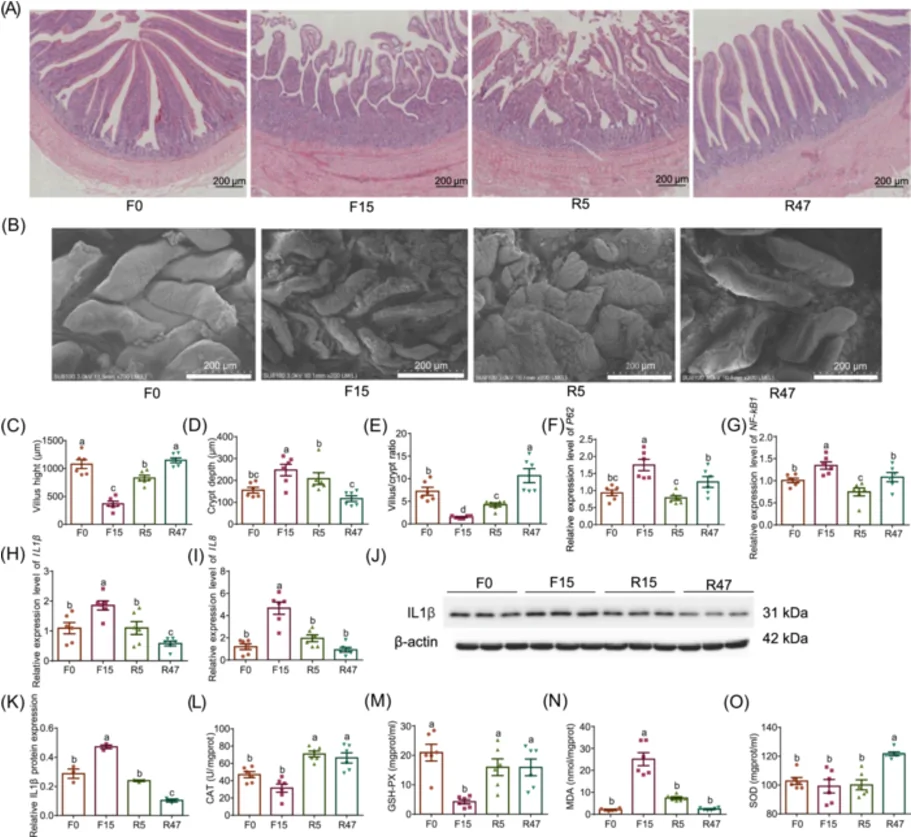
Changes in morphological structure, inflammatory response, and oxidative damage in the intestine of laying hens during FIM. (A) H&E staining of jejunum, Scale bars: 200 μm, *n* = 6. (B) Transmission electron microscopy of jejunum, Scale bars: 200 μm, *n* = 6. (C) Villus height of jejunum, *n* = 6. (D) Crypt depth of jejunum, *n* = 6. (E) Villus/crypt ratio of jejunum, *n* = 6. (F–I) Jejunal mucosa mRNA expression of *P62* (F), *NF‐kB1* (G), *IL‐1β* (H), and *IL‐8* (I), *n* = 6. (J) Jejunal mucosa protein expression of IL‐1β, *n* = 3. (K) The relative protein expression levels of IL1β are during FIM. (L–O) The levels of Catalase (CAT) (L), Glutathione Peroxidase (GSH‐PX) (M), Malondialdehyde (MDA) (N), and Superoxide Dismutase (SOD) (O), *n* = 6. Data were shown as the mean ± SEM, data with different superscript letters (a, b, and c) were significantly different (*p* < 0.05).

To elucidate jejunal mucosal remodeling, we analyzed cell proliferation, differentiation, autophagy, and apoptosis during FIM. During fasting, we observed a significant upregulation of proliferation (*CCNB1*, *CDK1*, *DLL1*, *OLFM4*, *BMI1*, and *LGR5*) (Figure S3A–F) and autophagy (*BECLIN1*, *ERN1*, and *P53*) genes (*p* < 0.05) (Figure S3G–I). Concurrently, differentiation genes (*SLC2A2*, *SLC5A1*, and *CALB1*) (Figure S3J–L) and pro‐apoptotic genes (*TNF‐α*, *FASL*, and *Caspase‐3*) were downregulated (Figure S3M–O), while anti‐apoptotic genes (*PDGFR* and *BCL2*) increased (*p* < 0.05) (Figure S3P,Q). Refeeding consistently reversed these mRNA expression patterns for all tested genes. Importantly, protein expression of key markers (CDK1, LGR5, SLC2A2, SLC5A1, CALB1, BCL2, and BECLIN1) mirrored their mRNA changes (Figure S4A–I). Additionally, LC3II/LC3I protein levels, elevated during fasting, significantly decreased upon refeeding (*p* < 0.05). Additionally, LC3II/LC3I protein levels, elevated during fasting, significantly decreased upon refeeding (*p* < 0.05) (Figure S4G,J). IF staining indicated that fasting significantly increased Ki67 proliferating stem cells (Figure S5A) while reducing the epithelial expression of SLC2A2, SLC5A1, and CALB1, which subsequently recovered to baseline levels following refeeding (Figure S5B–D). FIM dynamically reshaped jejunal mucosa by concurrently inducing intestinal stem cell activation and autophagy‐mediated villus atrophy during fasting and followed by rapid regeneration and repair upon refeeding.

### 2.3 RNA‑seq analysis of the mechanism underlying intestinal mucosa remodeling

To elucidate intestinal mucosa injury‐repair mechanisms during FIM, jejunal samples underwent RNA‐seq analysis. PCA, Venn diagram, and multiple scatter plots revealed significant genetic differences across time points (Figure 3A–C). WGCNA of jejunum transcriptome data, integrating villus height, crypt depth, and villus/crypt ratio (Figure S6A,B), identified blue, green‐yellow, yellow, and red modules as vital for intestinal villi regeneration (Figure 3D; Figure S6C–G). The hub genes from the blue, green‐yellow, yellow, and red modules were collectively integrated for GO and KEGG analyses. GO analysis revealed that hub genes were notably enriched in tissue development, including epithelial development, circulatory system development, and cell proliferation (Figure 3E; Table S3). Subsequent KEGG enrichment analysis revealed that hub genes were notably enriched in signaling pathways, including TGF‐beta signaling pathways (Figure 3F), which were closely stem cell proliferation and differentiation and inflammatory response. Activation of the TGF‐β signaling pathway during fasting was collectively indicated by heatmap analysis of key TGF‐β signaling gene counts, qRT‐PCR, and Western Blotting (Figure 3G–L; Figure S6H–S).

**Figure 3:**
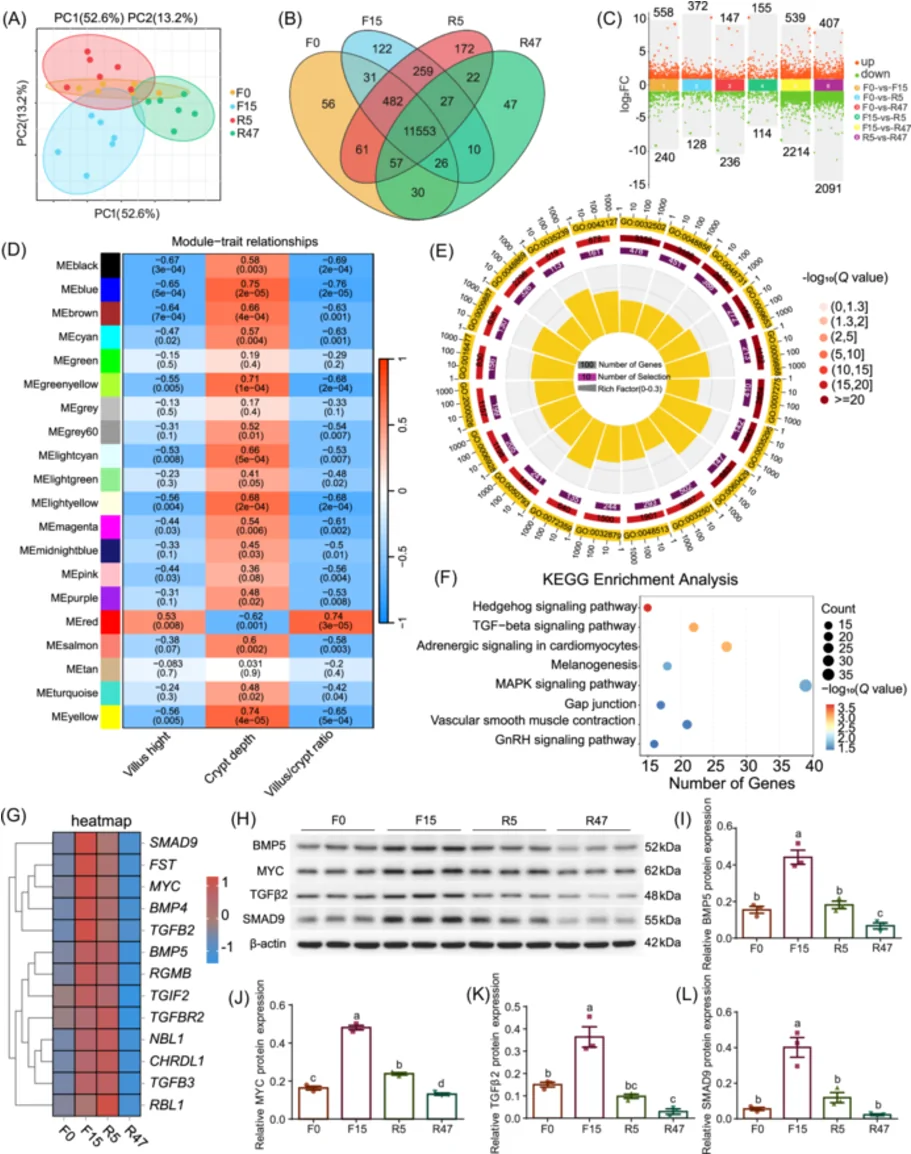
Analysis of the jejunal transcriptome data during FIM. (A) PCA analysis, *n* = 6. (B) Difference Venn diagram of the relative comparison group. (C) Multiple scatter plot analysis was conducted to assess the gene expression variances among samples at various time points, *n* = 6. (D) Analysis of correlations between the modules and villus height, crypt depth, and villus/crypt ratio, *n* = 6. (E) Circular visualization of GO enrichment analysis. Each sector represents a significantly enriched GO term (outer yellow ring), with the outer scale indicating gene counts (log_10_ scale). Purple bars and labels denote the number of enriched genes per term, while the red gradient reflects enrichment significance (−log_10_ *Q* value), with darker colors indicating higher significance. The inner bars represent the rich factor. (F) KEGG analysis of key genes. (G) RNA‐seq count data for the TGF‐beta signaling pathway genes in the jejunal mucosa, *n* = 6. (H) Jejunal mucosa protein expression of BMP5, MYC, TGF β2, and SMAD9. (I–L) The relative protein expression levels of BMP5 (I), MYC (J), TGF β2 (K), and SMAD9 (L) during FIM, *n* = 3. Data were shown as the mean ± SEM, data with different superscript letters (a, b, and c) were significantly different (*p* < 0.05).

### 2.4 Changes in liver function during FIM

To understand the impact of FIM on liver function, we performed liver transcriptome sequencing, revealing dynamic gene expression patterns (Figure 4A–C). Series test of cluster analysis was performed on the differentially expressed genes identified from the liver transcriptome. Genes from modules 2 and 5, which showed similar expression patterns, were selected for further functional annotation (Figure S7). GO and KEGG enrichment analyses indicated that the liver underwent metabolic reprogramming during fasting, accompanied by enhanced detoxification processes and disturbances in bile acid metabolism (Figure 4D,E; Table S4). Liver function assessment showed fasting significantly increased serum aspartate aminotransferase (AST), aspartate aminotransferase/alanine aminotransferase ratio (AST/ALT), total bile acids (TBA), and total bilirubin (TBIL‐Z), while decreasing albumin (ALB) (Figure 4F–J), indicating impaired liver function and bile acid accumulation [15]. Targeted metabolomics of liver and serum bile acids confirmed an overall increase during fasting (Figure S8A,B), with specific elevations of taurodeoxycholic acid (TDCA), taurolithocholic acid (TLCA), ursodeoxycholic acid (UDCA), tauroursodeoxycholic acid (TUDCA), and lithocholic acid (LCA), which then decreased upon refeeding (Figure 4K–T). qRT‐PCR revealed fasting downregulated *CYP7A1* and upregulated *CYP46A1*, *CYP7B1*, and *CYP27A1* (Figure S8C–F), suggesting activation of non‐classical bile acid synthesis pathways [16, 17].

**Figure 4:**
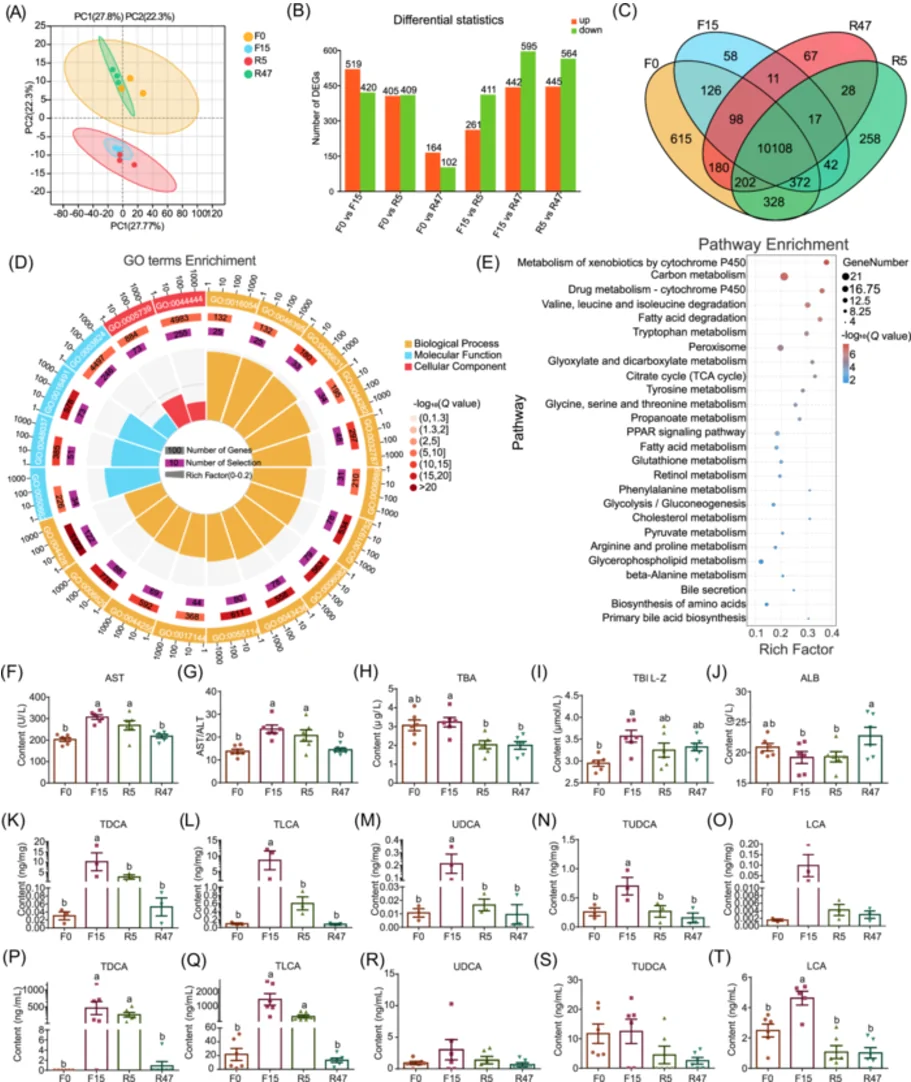
Changes in liver function during FIM. (A) PCA analysis, *n* = 3. (B) Overall statistics of significantly different genes, *n* = 3. (C) Difference Venn diagram of the relative comparison group. (D) GO analysis. (E) KEGG analysis. (F–J) Liver function tests include aspartate aminotransferase (AST) (F), aspartate aminotransferase/alanine aminotransferase ratio (AST/ALT) (G), total bile acids (TBA) (H), total bilirubin (TBIL‐Z) (I), and albumin (ALB) (J), *n* = 6. (K–O) Detections of liver bile acid concentrations include taurodeoxycholic acid (TDCA) (K), taurolithocholic acid (TLCA) (L), ursodeoxycholic acid (UDCA) (M), tauroursodeoxycholic acid (TUDCA) (N), and lithocholic acid (LCA) (O), *n* = 3. (P–T) Detections of serum bile acid concentrations include ursodeoxycholic acid (UDCA) (P), taurolithocholic acid (TLCA) (Q), ursodeoxycholic acid (UDCA) (R), tauroursodeoxycholic acid (TUDCA) (S), and lithocholic acid (LCA) (T), *n* = 6. Data were shown as the mean ± SEM, data with different superscript letters (a, b, and c) were significantly different (*p* < 0.05).

### 2.5 Integration analysis of cecal microbiota impact on intestinal and liver health

To investigate the relationship between changes in gut–iver health and the metabolites, we conducted a combined analysis of cecal metabolites with the transcriptomes of the jejunum and liver. Shared pathways between cecal metabolites and jejunum transcriptomes included ABC transporters, arachidonic acid metabolism, primary bile acid biosynthesis, and pantothenate and CoA biosynthesis (Figure 5A). This analysis identified a total of 53 key metabolites, including l‐serine, arachidonic acid (AA), LCA, D‐panthenol, and 3‐hydroxyanthranilic acid (3‐HAA) (Figure S9A). Association analysis results revealed that l‐serine, LCA, and AA showed positive relationships with TGF‐beta signaling and intestinal injury, while d‐panthenol and 3‐HAA exhibited opposing effects (Figure 5B). Similarly, common pathways enriched by cecal metabolites and liver transcriptomes included ABC transporters, primary bile acid biosynthesis, arachidonic acid metabolism, and pantothenate and CoA biosynthesis (Figure 5C). This analysis identified a total of 56 key metabolites, including l‐serine, AA, D‐panthenol, LCA, and 3‐HAA (Figure S9B). The association analysis results revealed that l‐serine, LCA, and AA were closely associated with liver injury, whereas D‐Panthenol and 3‐HAA played important roles in liver function repair (Figure 5D).

**Figure 5:**
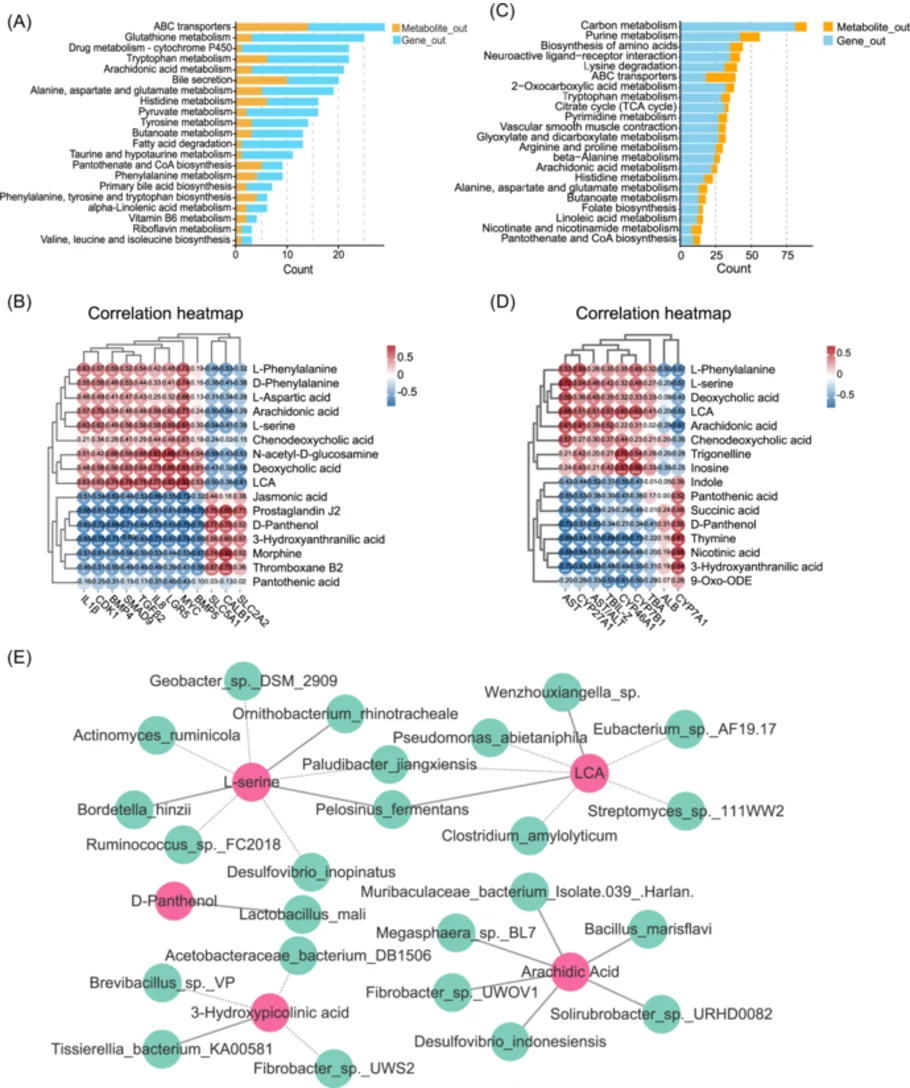
Multi‐omics integration analysis. (A) Analysis of the common pathways enriched by cecal metabolite profiles and the jejunal transcriptome. (B) The correlation heatmap of intestinal injury repair and cecal metabolites. (C) Analysis of the common pathways enriched by cecal metabolite profiles and the liver transcriptome. (D) The correlation heatmap of liver function and cecal metabolites. Significance levels were defined as follows: * for 0.01 < *p* < 0.05, ** for 0.001 < *p* < 0.01, and *** for *p* < 0.001. (E) PPI of maaslin2 analysis of cecal microbiome and metabolome. Green represented microbiome, purple represented metabolites, full line represented a positive correlation, and dotted line represented a negative correlation.

To investigate associations between intestinal metabolites and the gut microbiota, we applied MaAsLin2 to perform multivariable linear modeling with confounder adjustment. Notable associations (|coef| > 2 and *q* < 0.05) include: *Liquorilactobacillus mali* is positively independent associated with D‐Panthenol; *Tissierellia* bacterium KA00581 is positively independent associated with 3‐HAA; *Pelosinus fermentans* is positively independent associated with l‐serine and with LCA; *Fibrobacter* sp. UWOV1 is positively independent associated with AA (Figure 5E; Table S5). Based on this, we hypothesized that *Liquorilactobacillus mali* and *Tissierellia* bacterium KA00581 facilitate the functional remodeling of both the gut and liver, while *Pelosinus fermentans* and *Fibrobacter* sp. UWOV1 was associated with impaired gut‐liver function during fasting.

### 2.6 d‐Panthenol promotes the growth of chicken small intestinal organoids

Based on our transcriptomic and metabolomic analyses, we investigated the effects of D‐Panthenol on intestinal function using a chicken small intestinal organoid model. Organoids were cultured with 0, 10, 100, and 1000 µM D‐Panthenol. By Day 3, budding was observed in all groups, and by Day 5, the number of buds and the organoid area had increased (Figure 6A).

**Figure 6:**
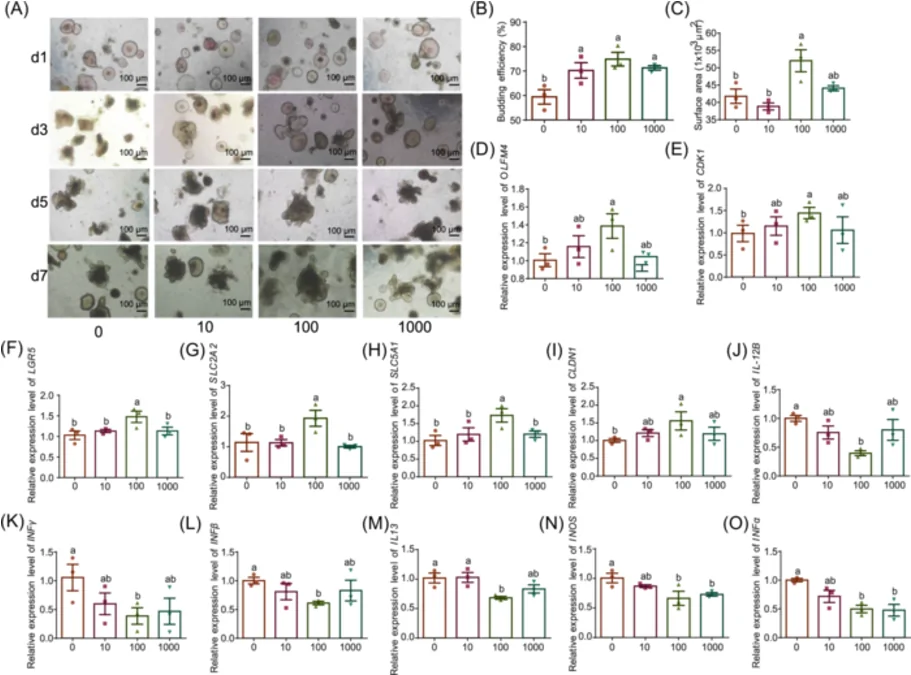
The effect of d‐Panthenol on the growth of chicken intestinal organoids. (A) Growth status of chicken intestinal organoids under different concentrations of d‐Panthenol at Days 1, 3, 5, and 7. (B) Budding rate of intestinal organoids at Day 7 across different d‐Panthenol concentrations. (C) Area of intestinal organoids at Day 7 under varying concentrations of d‐Panthenol. The expression level of *OLFM4* (D), *CDK1* (E), *LGR5*(F), *SLC2A2* (G), *SLC5A1* (H), *CLDN1* (I), *IL12B* (J), *INFγ* (K), *IFNβ* (L), *IL13* (M), *INOS* (N), and *INFα* (O). Data were shown as the mean ± SEM, data with different superscript letters (a, b, and c) were significantly different (*p* < 0.05), *n* = 3. Scale bars: 100 μm.

By Day 7, compared to controls, the budding rates in the 10, 100, and 1000 µM groups were significantly higher (*p* < 0.05) (Figure 6B), with the 100 µM group showing the most significant increase in area (*p* < 0.05) (Figure 6C). qRT‐PCR analysis on Day 7 revealed 100 µM D‐Panthenol significantly upregulated proliferation (*OLFM4*, *CDK1*, and *LGR5*), differentiation (*SLC2A2* and *SLC5A1*), and barrier function (*CLDN1*) genes (*p* < 0.05) (Figure 6D–I). Concurrently, inflammation‐related genes (*IL12B*, *INFγ*, *IFNβ*, *IL13*, *iNOS*, and *INFα*) were significantly reduced (*p* < 0.05) (Figure 6J–O). These results indicate that 100 µM D‐Panthenol promotes the proliferation and differentiation of small intestinal organoids, enhances their barrier function, and mitigates inflammation.

## 3 DISCUSSION

FIM is an approach to reactivate the production performance of laying hens, but it involves a period of fasting that can lead to energy deficiency. This energy deficit disrupts the gut microbiota composition and compromises gut–liver functionality, ultimately contributing to higher mortality rates. Consistent with previous studies, our data demonstrate that fasting enhances pathogenic bacterial abundance, consequently increasing the gut′s susceptibility to invasion [6, 18]. During fasting, *Candidatus Aminicenantes* noname, *Enterobacter*, and *Slackia* become predominant genera. We hypothesize that these bacteria have the ability to ferment and utilize undigested carbohydrates. Notably, some members also exhibit pathogenic potential; for example, *Enterobacter species* can readily cause infections when host immunity is compromised or the physical barrier is disrupted [19]. After refeeding, probiotic levels increase, improving the adaptability of the gut microbiome and playing a crucial role in energy metabolism, host health regulation, and the maintenance of microbial ecological balance. During R5, *Ligilactobacillus salivarius* and *Bacteroides uniformis* were clustered, contributing to the maintenance of the gut microbiota balance and the regulation of anti‐inflammatory responses [20, 21, 22, 23]. During R47, *Butyricicoccus porccorum* were clustered, potentially influencing the growth and renewal of intestinal epithelial cells through the production of beneficial short‐chain fatty acids [24]. Although metagenomic sequencing reveals subtle shifts in the gut microbiota during FIM, knowledge of the specific functions of most involved microbes remains limited.

Metabolites act as a crucial bridge between gut microbiota and host health. The shifting correlation patterns (e.g., SA and N‐acetyl‐Asp‐Glu prominence at F15; 4‐(1‐adamantyl)‐2‐methyl‐1,3‐thiazole prominence at R47) point to a dynamic reprogramming of metabolic routes likely reflecting changes in mucosal immunity, barrier function, or energy metabolism as the condition evolves. The increase in compounds such as SA during F15 concurrently implies adaptive or protective responses by the body to manage fasting stress, regulate inflammation, or preserve homeostasis [25]. During fasting, the enrichment of metabolic pathways involved in secondary bile acid metabolism indicates substantial gut and liver functional impairment. Bile acid accumulation not only damages liver cells and triggers inflammation but also impairs intestinal barrier function by disrupting enterohepatic circulation, which can subsequently lead to injury of the intestinal mucosa [26, 27]. While KEGG identifies metabolite functions, direct physiological impact requires further investigation. Thus, we analyzed intestinal and liver function changes to clarify metabolite roles during FIM.

Consistent with previous studies, the intestine underwent dynamic changes during FIM, transitioning from inflammation and oxidative damage to repair [6]. Uniquely, our study found increased proliferative capacity of intestinal cells during fasting in hens, followed by rapid differentiation of intestinal stem cells upon refeeding. In a study involving mice, fasting for 24 h significantly reduced the proliferation of crypt cells, whereas refeeding enhanced intestinal cell proliferation [28]. We hypothesize that hens, with higher fat reserves from late laying, utilize fat autophagy during fasting to provide essential nutrients [29, 30], thus preserving intestinal stem cell regenerative and repair capacities. In addition, we found that TGF‐beta signaling pathways are activated during fasting. The TGF‐beta signaling pathway plays a central role in regulating inflammatory responses, controlling autophagy, and modulating cell proliferation, all of which are essential for maintaining tissue homeostasis and promoting tissue repair [31, 32]. This leads us to speculate that the TGF‐beta pathway is a critical regulator of intestinal mucosal remodeling.

FIM profoundly impacts liver function due to significant nutritional and energy fluctuations [9]. During fasting, the concentrations of bile significantly increased in both the liver and serum samples, along with the activation of non‐classical bile acid synthesis pathways in the liver. Under physiological conditions, the classical pathway converts cholesterol to 7α‐hydroxycholesterol via CYP7A1, resulting in the production of bile acids CA and CDCA. In contrast, the alternative pathway is initiated by CYP27A1 and generates CDCA through 7α‐hydroxylation catalyzed by CYP7B1, with CYP8B1 also playing a role in bile acid hydroxylation [33]. This study found that fasting suppresses the expression of CYP7A1 while upregulating CYP27A1 and CYP7B1, indicating that fasting activates the non‐classical pathway of bile acid synthesis. The activation of this pathway during fasting leads to the synthesis of bile acids, which help regulate energy balance in response to altered metabolic demands [33]. However, this activation can promote abnormal bile acid accumulation, impair bile excretion, and cause liver cell damage [34], disrupting cellular integrity and promoting inflammation and fibrosis, which may ultimately lead to cirrhosis. High concentrations of bile acids can be toxic to liver cells, causing damage to cell membranes, leading to cell death, and subsequently affecting liver function [35]. If the issue of bile acid accumulation is not resolved, this damage may have a negative impact on overall health, prolong the recovery time during FIM, reduce the increase in egg production rates, and increase mortality rates.

A comprehensive examination of the gut‐liver function is essential for understanding metabolism, immunity, and disease mechanisms [36, 37]. Focusing solely on either the gut or liver neglects their intricate interactions, hindering a full understanding of how fasting impacts overall health. MaAsLin2 analysis revealed *Liquorilactobacillus mali* to be independently and positively associated with D‐Panthenol, and *Tissierellia* bacterium KA00581 to be independently and positively associated with 3‐HAA. Both of these metabolites are implicated in gut and liver repair processes. d‐Panthenol and 3‐HHA may have positive effects on intestinal and liver functions through their antioxidant and cell‐protective effects [38, 39, 40]. Our study demonstrated 100 µM D‐Panthenol significantly promoted chicken small intestinal organoid proliferation and differentiation, and enhanced barrier function by increasing *CLDN1* and reducing inflammation [41]. Thus, D‐Panthenol may serve as a valuable exogenous additive to improve gut health in laying hens during FIM. Furthermore, *Pelosinus fermentans* showed independent positive associations with l‐serine and LCA, while *Fibrobacter* sp. UWOV1 was independently and positively associated with AA. These findings suggest their involvement in gut and liver damage. Elevated levels of LCA and AA are positively correlated with metabolic disorders in the gut and with liver inflammation [42, 43, 44]. Conversely, increased l‐serine metabolism exerts beneficial effects on both the gut and liver [45, 46]. We hypothesized that this dysfunction enhances l‐serine metabolism, thereby reducing the damage to the gut and liver. Correlation analysis of gut microbiota, metabolites, and gut‐liver function offers insights into their roles, but does not establish causality. Further experimental validation is required to confirm the relationships mentioned above.

## 4 CONCLUSIONS

The study investigated the dynamic processes of the gut microbiota, metabolites, intestinal remodeling, and liver function during FIM. During fasting, harmful microbiota and metabolites increased, causing oxidative damage, intestinal inflammation, and villus atrophy. The process activated the TGF‐beta signaling pathway, promoting intestinal stem cell proliferation. Moreover, liver function dysfunction, and non‐classical bile acid synthesis was activated, causing bile acid accumulation in the liver. During refeeding, the previously observed effects were reversed. We identified key microorganisms (*Liquorilactobacillus mali* and *Tissierellia* bacterium KA00581) and functional metabolites (d‐Panthenol and 3‐HAA) associated with intestinal‐liver function during FIM. This study provides evidence at the organoid level that d‐Panthenol can serve as a candidate exogenous additive to improve intestinal health in laying hens undergoing FIM. This study elucidated the correlations among the gut microbiota, metabolites, and gut–liver function, offering guidance for enhancing FIM management and feeding strategies to prolong the lifespan of laying hens.

## 5 METHODS

### 5.1 Animals and experimental design

A total of 90 laying hens, aged 60 weeks with similar body weights, were divided into 9 replicates of 10 hens each. The study was conducted at the Poultry Germplasm Resource Factory of Henan Agricultural University in Yuan Yang County, Henan Province, from April to July 2022. The chickens were maintained in a room with a temperature of 18°C–25°C and humidity of 60–70%. All chickens were fed a corn‐soybean meal basal diet [47]. The FIM experiment was focused on four time points: F0, F15, R5, and R47 (Figure S10). In this study, the weight loss during F15 was maintained below 30%, and the second peak of egg production occurred during R47.

### 5.2 Metagenomic sequencing and analysis

Metagenomic sequencing data were deposited in the Genome Sequence Archive under accession number CRA021343 [7]. This gene set was aligned and annotated against various databases, including KEGG, for functional analysis of the microbial community. Inter‐group comparisons of Shannon diversity were performed using the Kruskal‐Wallis test. PCoA based on Bray–Curtis dissimilarities at various taxonomic levels was employed to visualize inter‐sample relationships. To identify species significantly contributing to community differentiation, LEfSe was performed [48]. LEfSe combines the non‐parametric Kruskal–Wallis test with linear discriminant analysis (LDA) to estimate the effect size of differentially abundant features among groups (*q* < 0.05 and LDA score > 3). The sequencing and bioinformatics analysis were performed using the Omicsmart online platform (http://www.omicsmart.com).

### 5.3 Cecal contents metabolite analysis

The frozen cecal contents were thawed on ice and extracted using an 80% methanol buffer. The extracted samples were subsequently analyzed by liquid chromatography‐mass spectrometry according to the manufacturer's instructions. Significantly differentiated metabolites were identified by combining PLS‐DA VIP scores with BH‐FDR corrected one‐way analysis of variance (ANOVA) results. The significance thresholds were set at VIP ≥ 1 and *q* < 0.05. Differentially expressed metabolites were visualized using a volcano plot and subjected to KEGG enrichment analysis. The sequencing and bioinformatics analysis were performed using the Omicsmart online platform (http://www.omicsmart.com).

### 5.4 Random forest feature selection and SparCC‐based correlation network analysis

Random forest analysis identified the top 50 significant gut microbiota and metabolites by Mean Decrease Gini importance, with statistical significance evaluated via permutation tests (*n* = 200) and BH‐FDR correction (*q* < 0.05) [49]. Raw abundance data were CLR‐transformed and regressed against covariates (year and fasting status) to obtain residuals. SparCC correlations were calculated from these residuals, with robust correlations (|*R*| > 0.7, *q* < 0.05) visualized via heatmaps [50]. Analyses were conducted in R (version 4.5.2) using the compositions (version 2.0‐9) and psych (version 2.6.1) packages.

### 5.5 H&E staining and immunofluorescence staining

Intestinal tissue samples were fixed in paraffin and cut into 4‐μm sections with a microtome (Leica Biosystems). Each section was then stained with H&E. Ten villi with intact linear structures were selected from the stained sections. Villus height and crypt depth were measured using CaseViewer software. The mean of these measurements was recorded for each sample, and the villus/crypt ratio was calculated. Jejunal tissue sections were prepared for immunofluorescence. The sections were subsequently incubated with primary antibodies (Ki67 (1:400), SLC2A2 (1:200), SLC5A1 (1:100), and CALB1 (1:200)) at 4°C overnight. The antibody information is shown in Table S6. Following incubation, the cells were rinsed with phosphate‐buffered saline with Tween (PBST) and treated with secondary antibodies (1:200) for 60 min at room temperature. Stained sections were visualized under a confocal microscope.

### 5.6 Scanning electron microscopy

Jejunum samples were fixed in 2.5% glutaraldehyde and incubated with 1% osmium tetroxide for 2 h at 4°C. They were dehydrated in an increasing ethanol gradient for 20 min at each step and then immersed in a 1:1 mixture of ethanol and tert‐butanol for 30 min. The samples were then incubated in tert‐butanol for 1 h. Dehydration was completed using a Hitachi Model HCP‐2 critical point dryer (Hitachi Koki) with liquid CO_2_. Finally, the dried samples were coated with gold‐palladium and analyzed using digital SEM (Hitachi S‐4800).

### 5.7 qRT‐PCR analysis

High‐quality RNA was reverse transcribed into cDNA using the Prime Script RT Reagent Kit (TaKaRa), followed by qRT‐PCR using a Light Cycler 96 system (Roche) and the SYBR Premix Ex Taq II kit (Takara). The primer sequences for these genes are shown in Table S7. qRT‐PCR was performed to analyze the mRNA levels of the reference gene using the 2^−ΔΔCT^ method.

### 5.8 Western blotting

Total protein extracts from the jejunum tissue samples were collected and quantified using the Bradford assay. The specific operational steps for the western blotting were based on the instructions provided by Pillai‐Kastoori et al. [51]. The antibody information is shown in Table S6. The expression of β‐actin was used as an internal loading control, and the optical density of protein bands was measured using ImageJ software.

### 5.9 Constructing cDNA library and sequence data analysis

Jejunum and liver tissue RNA was extracted using TRIzol reagent and quantified using a NanoDrop ND‐2000 spectrophotometer (Thermo Fisher Scientific), and integrity was assessed via agarose gel electrophoresis. High‐quality RNA samples were selected for library preparation and sequenced using an Illumina NovaSeq. 6000 platform (illumina). Quality control of raw sequencing reads was conducted using FASTQ by removing reads with adapters, high N ratios, or low quality, resulting in high‐quality clean reads. These clean reads were aligned to the GRCg7b reference genome using HISAT2, and the transcripts were assembled using StringTie software. Gene expression was quantified using RNA‐Seq by Expectation‐Maximization to obtain raw counts. For downstream differential expression analysis and gene expression comparisons, raw counts were used. Differential expression analysis was performed using DESeq. 2 (version 1.30.0) with the following criteria: |log_2_Fold‐Change| > 1 and *q* < 0.05 [52]. The jejunal transcriptome sequencing analyses were performed using the Omicsmart online platform (http://www.omicsmart.com). The liver transcriptome sequencing analyses were performed using the Majorbio Cloud (www.majorbio.com).

### 5.10 Weighted gene co‐expression network analysis (WGCNA)

WGCNA employs co‐expression analysis to uncover gene interactions linked to traits (villus height, crypt depth, and villus/crypt ratio), thereby elucidating the complex interplay between gene expression and these traits. Using the “WGCNA” package in R (version 4.5.2) [53], the training set genes were networked with a soft threshold function ensuring a correlation coefficient |*R*| > 0.80 for a scale‐free network. Module correlations between traits were calculated and visualized using heatmaps. GO and KEGG pathway enrichment analyses of gene significance were conducted using the “clusterProfiler” package in R and the DAVID database.

### 5.11 Series test of cluster analysis and functional enrichment

To characterize dynamic transcriptional responses in liver tissues during FIM, time‑series clustering analysis was performed in R (version 4.5.2). Gene expression data were first log_2_‑transformed, and genes with low expression were removed to reduce noise. The remaining genes were standardized using Z‑score transformation to ensure comparable expression patterns across samples. Temporal expression patterns were identified using the K‑means clustering algorithm. The optimal number of clusters was determined based on the elbow method by evaluating the within‑cluster sum of squares across different cluster numbers. Genes were then assigned to clusters representing distinct temporal expression trends across the four time points (F0, F15, R5, and R47). For each cluster, the average standardized expression profile was calculated to represent the characteristic temporal trajectory of that module. To further evaluate clustering reliability, fuzzy c‑means clustering implemented in the e1071 package was applied to estimate membership scores for each gene. Genes with higher membership values were considered to have stronger associations with their corresponding temporal patterns. Genes within each cluster were subsequently used for downstream functional enrichment analysis.

### 5.12 Detection of serum liver function indicators

Liver function indicators, including ALT, AST, ALP, ALB, TBA, GGT, TBIL‐Z, and DBIL‐Z were analyzed using a fully automated biochemical analyzer (BK280).

### 5.13 Targeted bile acid metabolomics sequencing

First, liver samples were weighed and ground for metabolite extraction at low temperature, while serum samples were mixed with methanol for extraction [54]. After centrifugation to obtain supernatants, both were analyzed using LC‐ESI‐MS/MS (UHPLC‐Qtrap) for qualitative and quantitative detection, with concentrations calculated via standard curves to quantify target substances.

### 5.14 Integration of genes or microbiota and metabolites

We explored the shared KEGG metabolic pathways involving genes and metabolites, evaluated their interactions, and constructed an integrated functional model. Correlations between genes and metabolites were analyzed by OmicShare tools (https://www.omicshare.com/tools/) [55]. We performed Maaslin2 analysis to assess the correlations between microorganisms and metabolites using the Maaslin2 package in R [56], and network analysis was performed using Cytoscape (version 3.8.2) [57].

### 5.15 Isolation and culture of chicken small elongated organoids

Small intestines from 18‐day‐old AA broiler embryos were dissected, opened longitudinally, and washed with PBS. Tissues were minced into 1–2 mm³ fragments and digested in 2.5 mmol/L EDTA at 4°C for 30 min. After vortexing for 30 s, crypt cells were filtered through a 100 μm strainer, centrifuged at 50 × *g* for 5 min, washed with PBS, and pelleted at 200 × *g* for 5 min. The pellet was resuspended in DMEM, mixed with undiluted Matrigel at a 2:1 ratio, and seeded into 24‐well plates (50 μL per well, 100–150 crypts per well). Following solidification at 37°C for 30 min, 500 μL of pre‐warmed complete medium was added per well. Complete medium was prepared by mixing l‐WRN‐conditioned medium and high‐glucose DMEM (1:1) supplemented with 10% fetal bovine serum, 1% penicillin/streptomycin, 1× B‐27, 1× N‐2, 1 mmol/L l‐glutamine, 10 mmol/L HEPES, 10 mmol/L nicotinamide, 500 ng/mL human R‐spondin 1, 100 ng/mL human Noggin, 50 ng/mL human EGF, 50 ng/mL FGF2, 1 μmol/L Jagged‐1, 3 μmol/L CHIR99021, 0.5 μmol/L galunisertib, 10 μmol/L SB202190, and 5 μmol/L Y‐27632. The medium was refreshed every 2 days. Primary organoids were passaged on Day 7 and subsequently every 5 days.

### 5.16 Statistical analysis

Statistical analyses were conducted using SPSS software (version 25.0). Group comparisons were evaluated using one‐way ANOVA followed by Tukey's post hoc test for normally distributed data, or the Kruskal–Wallis test for non‐normally distributed data. Each experiment was performed independently with at least three replicates to ensure reliability. Statistical significance was set at *p* < 0.05. The results are presented as the mean ± SEM. GraphPad Prism (version 8.0) was used to generate clear and comprehensive visual representations of the data.

## AUTHOR CONTRIBUTIONS

**Hao Zhang**: Writing—original draft; conceptualization; methodology; writing—review & editing; formal analysis; data curation; project administration; visualization. **Zhixuan Luo**: Methodology; validation. **Jun Zhang**: Methodology; validation. **Chenxu Wang**: Methodology; investigation; validation. **Yadong Tian**: Methodology; investigation; validation. **Donghua Li**: Formal analysis; investigation; methodology; validation; visualization. **Yujie Guo**: Methodology; investigation; validation. **Yanhua Zhang**: Methodology. **Wenting Li**: Methodology; investigation; validation. **Yujie Gong**: Formal analysis; methodology; investigation; validation; writing—review & editing. **Xiangtao Kang**: Conceptualization; validation; writing—review & editing; funding acquisition. **Ruirui Jiang**: Conceptualization; methodology; validation; writing—review & editing; funding acquisition. All authors have read the final manuscript and approved it for publication.

## CONFLICT OF INTEREST STATEMENT

The authors declare no conflicts of interest.

## ETHICS STATEMENT

All work involving animal experiments were approved by the Regulators for the Administration of Affairs Concerning Experimental Animals (Revised Edition, 2017). The experiments conducted in this study were approved by the Institutional Animal Care and Use Committee of Henan Agricultural University (permit number: HNND2023021501).

## AI STATEMENT

ChatGPT (version 5.2) was used exclusively for language polishing, including grammar correction and stylistic refinement. All content has been verified and confirmed by the authors as accurate.

## Supporting information


**Figure S1:** Key biological features of gut microbiome and metabolome during FIM.
**Figure S2:** Changes in morphological structure in the ileum of laying hens during FIM.
**Figure S3:** Jejunal mucosa mRNA expression of proliferation, autophagy, differentiation, and apoptotic genes.
**Figure S4:** Jejunal mucosa protein expression of proliferation, autophagy, differentiation, and apoptotic genes.
**Figure S5:** Immunofluorescence staining.
**Figure S6:** Analysis of the jejunal transcriptome data during FIM.
**Figure S7:** Series test of cluster analysis of differentially expressed genes in the liver transcriptome.
**Figure S8:** Detection of liver function and the expression of bile acid synthesis‐related genes.
**Figure S9:** Metabolite Heatmaps.
**Figure S10:** The timeline of FIM.

**Table S1:** Random forest analysis of microbial genera feature importance.
**Table S2:** Random forest analysis of metabolites feature importance.
**Table S3:** GO analyses of key genes.
**Table S4:** GO analysis of key genes in modules 2 and 5.
**Table S5:** Significant associations between intestinal microbiota genus and metabolites identified by MaAsLin2.
**Table S6:** Antibody information for Western blotting.
**Table S7:** The qRT‐PCR primers.

## Data Availability

The data that support the findings of this study are openly available in GSA at https://ngdc.cncb.ac.cn/gsa/, reference numbers CRA021254 and CRA021264. Metagenomic sequencing data from cecal contents, along with the RNA‐seq data from the jejunum and liver have been archived in the Genome Sequence Archive under the primary accession codes CRA021254 (https://ngdc.cncb.ac.cn/gsa/browse/CRA021254) and CRA021264 (https://ngdc.cncb.ac.cn/ gsa/browse/CRA021264). Other used data is provided within the manuscript or supplementary information files. The code used for data analysis and figure generation is available on GitHub (https://github.com/zhaohao2026/imetaomic). Supplementary materials (figures, tables, graphical abstract, slides, videos, Chinese translated version, and updated materials) may be found in the online DOI or iMeta Science http://www.imeta.science/imetaomics/.

## References

[1] Mishra, R. , B. Mishra , Y. S. Kim , and R. Jha . 2022. “Practices and Issues of Moulting Programs for Laying Hens: A Review.” British Poultry Science 63: 720–729. 10.1080/00071668.2022.2059339 35363112

[2] Berry, W. D . 2003. “The Physiology of Induced Molting.” Poultry Science 82: 971–980. 10.1093/ps/82.6.971 12817453

[3] Cooper, J . 1978. “Moult of the Black–Footed Penguin.” International Zoo Yearbook 18: 22–27. 10.1111/j.1748-1090.1978.tb00211.x

[4] Cherel, Y. , J. B. Charrassin , and E. Challet . 1994. “Energy and Protein Requirements for Molt in the King Penguin Aptenodytes Patagonicus.” American Journal of Physiology‐Regulatory, Integrative and Comparative Physiology 266: R1182–R1188. 10.1152/ajpregu.1994.266.4.R1182 8184961

[5] Zhang, Xiaoran , Jun Zhang , Yadong Tian , Donghua Li , Yanhua Zhang , Lujie Zhang , Yujie Gong , Xiangtao Kang , and Ruirui Jiang . 2025. “Fasting and Refeeding Mediated Phospholipid Remodeling Plays an Important Role in Improving Meat Quality of Aged Laying Hens.” Animal Research and One Health. In press. 10.1002/aro2.70036

[6] Zhang, Hao , Yihui Zhang , Yujie Gong , Jun Zhang , Donghua Li , Yadong Tian , Ruili Han , et al. 2024. “Fasting‐Induced Molting Impacts the Intestinal Health by Altering the Gut Microbiota.” Animals 14: 1640. 10.3390/ani14111640 PMC11171271 38891687

[7] Zhang, Hao , Chenxu Wang , Yujie Gong , Yidan Zhu , Mengyan Zhang , Donghua Li , Yadong Tian , et al. 2025. “Changes in Pathogenicity of Gut Microbiota During Fasting‐Induced Molting in Laying Hens and Their Impact on Spleen Immune Function.” Poultry Science 104: 105526. 10.1016/j.psj.2025.105526 PMC12356445 40784060

[8] Holt, P. S . 2003. “Molting and Salmonella Enterica Serovar Enteritidis Infection: the Problem and Some Solutions.” Poultry Science 82: 1008–1010. 10.1093/ps/82.6.1008 12817457

[9] Zhang, Jun , Xiaoqin Geng , Yihui Zhang , Xinlong Zhao , Pengwei Zhang , Guirong Sun , Wenting Li , et al. 2022. “Interaction Between Cecal Metabolites and Liver Lipid Metabolism Pathways During Induced Molting in Laying Hens.” Frontiers in Physiology 13: 862721. 10.3389/fphys.2022.862721 PMC9169092 35677092

[10] Zhang, Jun , Yujie Gong , Yidan Zhu , Qingduo Zeng , Hao Zhang , Ruili Han , Yujie Guo , et al. 2025. “Exploring the Metabolic Patterns and Response Mechanisms of Bile Acids During Fasting: A Study with Poultry As an Example.” Poultry Science 104: 104746. 10.1016/j.psj.2024.104746 PMC11770500 39799857

[11] Wang, Weiwei , Jing Wang , Haijun Zhang , Shugeng Wu , and Guanghai Qi . 2019. “Transcriptome Analysis Reveals Mechanism Underlying the Differential Intestinal Functionality of Laying Hens in the Late Phase and Peak Phase of Production.” BMC Genomics 20: 970. 10.1186/s12864-019-6320-y PMC6907226 31830910

[12] Anene, Doreen Onyinye , Yeasmin Akter , Peter John Groves , Neil Horadagoda , Sonia Yun Liu , Amy Moss , Christine Hutchison , and Cormac John O'Shea . 2023. “Association of Feed Efficiency With Organ Characteristics and Fatty Liver Haemorrhagic Syndrome in Laying Hens.” Scientific Reports 13: 5872. 10.1038/s41598-023-30007-1 PMC10090132 37041185

[13] Liu, Qiulin , Jiabo Li , Di Han , Jinfeng Wang , Jian Zheng , Wei Ma , Chunqiang, Wang . 2024. “N‐Carbamylglutamate Supplementation Induces Functional Egg Production in Layers by Modulating Liver Transcriptome Profiles.” Poultry Science 103: 103223. 10.1016/j.psj.2023.103223 PMC10698670 38035861

[14] Shi, Kai , Xiangping Liu , Ying Duan , Xusheng Jiang , Ni Li , Yuesong Du , Dongfeng Li , and Chungang Feng . 2024. “Dynamic Changes in Intestinal Gene Expression and Microbiota Across Chicken Egg‐Laying Stages.” Animals 14: 1529. 10.3390/ani14111529 PMC11171086 38891577

[15] Sánchez‐Morales, Germán , Emma Castro , Ismael Domínguez‐Rosado , Mario Vilatoba , Alan Contreras , and Miguel Mercado . 2022. “Postoperative Liver Function Tests Can Predict Anastomotic Dysfunction After Bile Duct Injury Repair.” Updates in Surgery 74: 937–944. 10.1007/s13304-022-01275-9 35415799

[16] Worthmann, Anna , Clara John , Malte C. Rühlemann , Miriam Baguhl , Femke‐Anouska Heinsen , Nicola Schaltenberg , Markus Heine , et al. 2017. “Cold‐Induced Conversion of Cholesterol to Bile Acids in Mice Shapes the Gut Microbiome and Promotes Adaptive Thermogenesis.” Nature Medicine 23: 839–849. 10.1038/nm.4357 28604703

[17] Huang, Fengjie , Xiaojiao Zheng , Xiaohui Ma , Runqiu Jiang , Wangyi Zhou , Shuiping Zhou , Yunjing Zhang , et al. 2019. “Theabrownin from Pu‐Erh Tea Attenuates Hypercholesterolemia Via Modulation of Gut Microbiota and Bile Acid Metabolism.” Nature Communications 10: 4971. 10.1038/s41467-019-12896-x PMC6823360 31672964

[18] Han, Gi Ppeum , Kyu‐Chan Lee , Hwan Ku Kang , Han Na Oh , Woo Jun Sul , and Dong Yong Kil . 2019. “Analysis of Excreta Bacterial Community after Forced Molting in Aged Laying Hens.” Asian‐Australasian Journal of Animal Sciences 32: 1715–1724. 10.5713/ajas.19.0180 PMC6817773 31480206

[19] Davin‐Regli, Anne , Jean‐Philippe Lavigne , and Jean‐Marie Pagès . 2019. “Enterobacter spp.: Update on Taxonomy, Clinical Aspects, and Emerging Antimicrobial Resistance.” Clinical Microbiology Reviews 32: e00002–e00019. 10.1128/cmr.00002-19 PMC6750132 31315895

[20] Li, Congxin , Kaixin Peng , Siqi Xiao , Yuanyuan Long , and Qin Yu . 2023. “The Role of Lactobacillus in Inflammatory Bowel Disease: From Actualities to Prospects.” Cell Death Discovery 9: 361. 10.1038/s41420-023-01666-w PMC10541886 37773196

[21] Yao, Mingfei , Yanmeng Lu , Ting Zhang , Jiaojiao Xie , Shengyi Han , Shuobo Zhang , Yiqiu Fei , et al. 2021. “Improved Functionality of Ligilactobacillus Salivarius Li01 in Alleviating Colonic Inflammation by Layer‐By‐Layer Microencapsulation.” npj Biofilms and Microbiomes 7: 58. 10.1038/s41522-021-00228-1 PMC8270932 34244520

[22] Dai, Wei , Jiaxue Zhang , Lu Chen , Junhong Yu , Junyi Zhang , Hua Yin , Qingsen Shang , and Guangli Yu . 2023. “Discovery of Bacteroides Uniformis F18‐22 as a Safe and Novel Probiotic Bacterium for the Treatment of Ulcerative Colitis From the Healthy Human Colon.” International Journal of Molecular Sciences 24: 14669. 10.3390/ijms241914669 PMC10572632 37834117

[23] Renouf, Mathieu , and Suzanne Hendrich . 2011. “Bacteroides Uniformis Is a Putative Bacterial Species Associated With the Degradation of the Isoflavone Genistein in Human Feces.” The Journal of Nutrition 141: 1120–1126. 10.3945/jn.111.140988 21525249

[24] Chang, Shihchang , Minghung Shen , Chihyi Liu , Chiming Pu , Jeming Hu , and Chijung Huang . 2020. “A Gut Butyrate‑Producing Bacterium *Butyricicoccus pullicaecorum* Regulates Short‑Chain Fatty Acid Transporter and Receptor to Reduce the Progression of 1,2‑Dimethylhydrazine‑associated Colorectal Cancer.” Oncology Letters 20: 327. 10.3892/ol.2020.12190 PMC7577080 33101496

[25] Li, Dan , Tiantian Xie , Tianyi Guo , Zuomin Hu , Mengyuan Li , Yanqin Tang , Qi Wu , et al. 2023. “Sialic Acid Exerts Anti‐Inflammatory Effect Through Inhibiting MAPK‐NF‐κB/AP‐1 Pathway and Apoptosis in Ulcerative Colitis.” Journal of Functional Foods 101: 105416. 10.1016/j.jff.2023.105416

[26] Chen, Yiqiu , Zhaoyu Yang , Jingchun Bai , Xinyu Wang , Qiongyu Yuan , Yuling Mi , and Caiqiao Zhang . 2024. “Bioactive Lignan Honokiol Alleviates Ovarian Oxidative Stress in Aging Laying Chickens by Regulating SIRT3/AMPK Pathway.” Antioxidants 13: 377. 10.3390/antiox13030377 PMC10967992 38539910

[27] Ferrebee, Courtney B. , Jianing Li , Jamie Haywood , Kimberly Pachura , Brian S. Robinson , Benjamin H. Hinrichs , Rheinallt M. Jones , Anuradha Rao , and Paul A. Dawson . 2018. “Organic Solute Transporter α‐β Protects Ileal Enterocytes From Bile Acid‐Induced Injury.” Cellular and Molecular Gastroenterology and Hepatology 5: 499–522. 10.1016/j.jcmgh.2018.01.006 PMC6009794 29930976

[28] Imada, Shinya , Saleh Khawaled , Heaji Shin , Sven W. Meckelmann , Charles A. Whittaker , Renan Oliveira Corrêa , Chiara Alquati , et al. 2024. “Short‐Term Post‐Fast Refeeding Enhances Intestinal Stemness Via Polyamines.” Nature 633: 895–904. 10.1038/s41586-024-07840-z PMC12103248 39169180

[29] Sun, Congjiao , Fangren Lan , Qianqian Zhou , Xiaoli Guo , Jiaming Jin , Chaoliang Wen , Yanxin Guo , et al. 2024. “Mechanisms of Hepatic Steatosis in Chickens: Integrated Analysis of the Host Genome, Molecular Phenomics and Gut Microbiome.” Gigascience 13: giae023. 10.1093/gigascience/giae023 PMC11152177 38837944

[30] Hu, Hong , Ying Huang , Anjian Li , Qianhui Mi , Kunping Wang , Liang Chen , Zelong Zhao , et al. 2024. “Effects of Different Energy Levels in Low‐Protein Diet on Liver Lipid Metabolism in the Late‐Phase Laying Hens Through the Gut‐Liver Axis.” Journal of Animal Science and Biotechnology 15: 98. 10.1186/s40104-024-01055-y PMC11238517 38987834

[31] Deng, Ziqin , Tao Fan , Chu Xiao , He Tian , Yujia Zheng , Chunxiang Li , and Jie He . 2024. “TGF‐β Signaling in Health, Disease and Therapeutics.” Signal Transduction and Targeted Therapy 9: 61. 10.1038/s41392-024-01764-w.2 PMC10958066 38514615

[32] Hata, Akiko , and Ye‐Guang Chen . 2016. “TGF‐β Signaling from Receptors to Smads.” Cold Spring Harbor Perspectives in Biology 8: a022061. 10.1101/cshperspect.a022061 PMC5008074 27449815

[33] Zhuang, Tongxi , Xunjiang Wang , Zixuan Wang , Lihua Gu , Dawei Yue , Zhengtao Wang , Xiaohua Li , et al. 2025. “Biological Functions and Pharmacological Behaviors of Bile Acids in Metabolic Diseases.” Journal of Advanced Research 75: 779–792. 10.1016/j.jare.2024.11.003 PMC12789734 39522690

[34] Kunst, Roni F. , Dirk R de Waart , Frank Wolters , Suzanne Duijst , Esther W. Vogels , Isabelle Bolt , Joanne Verheij , et al. 2022. “Systemic ASBT Inactivation Protects against Liver Damage in Obstructive Cholestasis in Mice.” Journal of High Energy Physics Reports 4: 100573. 10.1016/j.jhepr.2022.100573 PMC9494276 36160754

[35] Li, Tiangang , Mohammad Nazmul Hasan , and Lijie Gu . 2024. “Bile Acids Regulation of Cellular Stress Responses in Liver Physiology and Diseases.” eGastroenterology 2: e100074. 10.1136/egastro-2024-100074 PMC11257078 39027418

[36] Pabst, Oliver , Mathias W. Hornef , Frank G. Schaap , Vuk Cerovic , Thomas Clavel , and Tony Bruns . 2023. “Gut‐Liver Axis: Barriers and Functional Circuits.” Nature Reviews Gastroenterology & Hepatology 20: 447–461. 10.1038/s41575-023-00771-6 37085614

[37] Albillos, Agustín , Andrea de Gottardi , and María Rescigno . 2020. “The Gut‐Liver Axis in Liver Disease: Pathophysiological Basis for Therapy.” Journal of Hepatology 72: 558–577. 10.1016/j.jhep.2019.10.003 31622696

[38] Said, Hamid M . 2011. “Intestinal Absorption of Water‐Soluble Vitamins in Health and Disease.” Biochemical Journal 437: 357–372. 10.1042/bj20110326 PMC4049159 21749321

[39] Gürler, Mukaddes , Engin Burak Selçuk , Beyza Güzide Özerol , Kevser Tanbek , Elif Taşlıdere , Azibe Yıldız , Fatma Hilal Yağın , and Elif Gürel . 2023. “Protective Effect of Dexpanthenol Against Methotrexate‐Induced Liver Oxidative Toxicity in Rats.” Drug and Chemical Toxicology 46: 708–716. 10.1080/01480545.2022.2084103 35655424

[40] Sabui, Subrata , Rubina Kapadia , Abhisek Ghosal , Michael Schneider , Nils W. G. Lambrecht , and Hamid M. Said . 2018. “Biotin and Pantothenic Acid Oversupplementation to Conditional SLC5A6 KO Mice Prevents the Development of Intestinal Mucosal Abnormalities and Growth Defects.” American Journal of Physiology‐Cell Physiology 315: C73–C79. 10.1152/ajpcell.00319.2017 PMC6087731 29669219

[41] Weber, Christopher R. , Sam C. Nalle , Maria Tretiakova , David T. Rubin , and Jerrold R. Turner . 2008. “Claudin‐1 and Claudin‐2 Expression Is Elevated in Inflammatory Bowel Disease and May Contribute to Early Neoplastic Transformation.” Laboratory Investigation 88: 1110–1120. 10.1038/labinvest.2008.78 PMC2586671 18711353

[42] Wang, Bei , Lujin Wu , Jing Chen , Lingli Dong , Chen, Chen , Zheng Wen , Jiong Hu , Ingrid, Fleming , Daowen Wang . 2021. “Metabolism Pathways of Arachidonic Acids: Mechanisms and Potential Therapeutic Targets.” Signal Transduction and Targeted Therapy 6: 94. 10.1038/s41392-020-00443-w PMC7910446 33637672

[43] Jang, Yongwoo , Minseok Kim , and Sun Wook Hwang . 2020. “Molecular Mechanisms Underlying the Actions of Arachidonic Acid‐Derived Prostaglandins on Peripheral Nociception.” Journal of Neuroinflammation 17: 30. 10.1186/s12974-020-1703-1 PMC6975075 31969159

[44] Cai, Jie , Sun Lulu , and Frank J. Gonzalez . 2022. “Gut Microbiota‐Derived Bile Acids in Intestinal Immunity, Inflammation, and Tumorigenesis.” Cell Host & Microbe 30: 289–300. 10.1016/j.chom.2022.02.004 PMC8923532 35271802

[45] Yang, Hong , Cheng Zhang , Hasan Turkez , Mathias Uhlen , Jan Boren , and Adil Mardinoglu . 2023. “Revisiting the Role of Serine Metabolism in Hepatic Lipogenesis.” Nature Metabolism 5: 760–761. 10.1038/s42255-023-00792-0 37169876

[46] Kitamoto, Sho , Christopher J. Alteri , Michael Rodrigues , Hiroko Nagao‐Kitamoto , Kohei Sugihara , Stephanie D. Himpsl , Malak Bazzi , et al. 2020. “Dietary L‐Serine Confers a Competitive Fitness Advantage to Enterobacteriaceae in the Inflamed Gut.” Nature Microbiology 5: 116–125. 10.1038/s41564-019-0591-6 PMC6925351 31686025

[47] Liang, Wenjie , Yuehua He , Tingqi Zhu , Binbin Zhang , Shuangxing Liu , Haishan Guo , Pingquan Liu , et al. 2024. “Dietary Restriction Promote Sperm Remodeling in Aged Roosters Based on Transcriptome Analysis.” BMC Genomics 25: 680. 10.1186/s12864-024-10544-3 PMC11232191 38978040

[48] Segata, Nicola , Jacques Izard , Levi Waldron , Dirk Gevers , Larisa Miropolsky , Wendy S. Garrett , and Curtis Huttenhower . 2011. “Metagenomic Biomarker Discovery and Explanation.” Genome Biology 12: R60. 10.1186/gb-2011-12-6-r60 PMC3218848 21702898

[49] Breiman, Leo . 2001. “Random Forests.” Machine Learning 45: 5–32. 10.1023/a:1010933404324

[50] Weiss, Sophie , Will Van Treuren , Catherine Lozupone , Karoline Faust , Jonathan Friedman , Ye Deng , Li Charlie Xia , et al. 2016. “Correlation Detection Strategies in Microbial Data Sets Vary Widely in Sensitivity and Precision.” The International Society for Microbial Ecology Journal 10: 1669–1681. 10.1038/ismej.2015.235 PMC4918442 26905627

[51] Pillai‐Kastoori, Lakshmi , Amy R. Schutz‐Geschwender , and Jeff A. Harford . 2020. “A Systematic Approach to Quantitative Western Blot Analysis.” Analytical BiochemistryAnal Biochem 593: 113608. 10.1016/j.ab.2020.113608 32007473

[52] Love, Michael I. , Wolfgang Huber , and Simon Anders . 2014. “Moderated Estimation of Fold Change and Dispersion for RNA‐seq Data With DESeq. 2.” Genome Biology 15: 550. 10.1186/s13059-014-0550-8 PMC4302049 25516281

[53] Langfelder, Peter , and Steve Horvath . 2008. “WGCNA: An R Package for Weighted Correlation Network Analysis.” BMC Bioinformatics 9: 559. 10.1186/1471-2105-9-559 PMC2631488 19114008

[54] Tian, Meng , Jun Yan , Honglong Zhang , Yuhui Wei , Mingtong Zhang , Zhi Rao , Mingkang Zhang , et al. 2022. “Screening and Validation of Biomarkers for Cadmium‐Induced Liver Injury Based on Targeted Bile Acid Metabolomics.” Environmental Pollution 300: 118837. 10.1016/j.envpol.2022.118837 35077840

[55] Mu, Hongyan , Jianzhou Chen , Wenjie Huang , Gui Huang , Meiying Deng , Shimiao Hong , Peng Ai , Chuan Gao , and Huangkai Zhou . 2024. “OmicShare Tools: A Zero‐Code Interactive Online Platform for Biological Data Analysis and Visualization.” Imeta 3: e228. 10.1002/imt2.228 PMC11488081 39429881

[56] Mallick, Himel , Ali Rahnavard , Lauren J. McIver , Siyuan Ma , Yancong Zhang , Long H. Nguyen , Timothy L. Tickle , et al. 2021. “Multivariable Association Discovery in Population‐Scale Meta‐Omics Studies.” PLoS Computational Biology 17: e1009442. 10.1371/journal.pcbi.1009442 PMC8714082 34784344

[57] Shannon, Paul , Andrew Markiel , Owen Ozier , Nitin S. Baliga , Jonathan T. Wang , Daniel Ramage , Nada Amin , Benno Schwikowski , and Trey Ideker . 2003. “Cytoscape: a Software Environment for Integrated Models of Biomolecular Interaction Networks.” Genome Research 13: 2498–2504. 10.1101/gr.1239303 PMC403769 14597658

