# Supplementary material for: Changes in the gut microbiome during fasting‐induced molting and its effects on gut–liver function
Source: IMetaOmics. 2026 May 20;3(2):e70103. doi: 10.1002/imo2.70103 (PMC13674437; doi:10.1002/imo2.70103)
Supplement: Supplementary file 1 [file IMO2-3-e70103-s002.docx]

Supporting information to

**Changes in The Gut Microbiome during Fasting-Induced Molting and Its Effects on Gut-Liver Function**

**Running title**: Effects of Gut Microbiota on Gut–Liver Function During Fasting-Induced Molting

Hao Zhang^1,2^, Zhixuan Luo^1^, Jun Zhang^1^, Chenxu Wang^1^, Yadong Tian^1^, Donghua Li^1^, Yujie Guo^1^, Yanhua Zhang^1^, Wenting Li^1,2^, Yujie Gong^1*^, Xiangtao Kang^1, 2*^, and Ruirui Jiang^1*^

^1^College of Animal Science and Technology, Henan Agricultural University, Zhengzhou, 450002, China

^2^The Shennong Laboratory, Zhengzhou Henan, 450002, China

***Corresponding author**: [gongyj1118@163.com](mailto:gongyj1118@163.com) (Yujie Gong); [xtkang2001@henau.edu.cn](mailto:xtkang2001@henau.edu.cn.net) (Xiangtao Kang); [jrrcaas@163.com](mailto:jrrcaas@163.com) (Ruirui Jiang).

**Figure S1.** **Key biological features of gut microbiome and metabolome during FIM.** (A) Dynamic trajectories and complex interactions of core microbiota. (B) PCoA analysis based on unweighted UniFrac metrics of microbial function. (C) Analysis of differences in the microbial function by LEfSe. (D) KEGG enrichment analysis of differential metabolites. (E) Dynamic trajectories and complex interactions of core metabolites. *n* = 6.

**Figure S2. Changes in morphological structure in the ileum of laying hens during FIM.** (A) H&E staining of ileum. (B) Villus height of ileum. (C) Crypt depth of ileum. (D) Villus/crypt ratio of ileum. Data were shown as the mean ± SEM, data with different superscript letters (a, b, and c) were significantly different (*p* < 0.05), Scale bars: 200 μm, *n* = 6.

**Figure S3.** **Jejunal mucosa mRNA expression of proliferation, autophagy, differentiation, and apoptotic genes.** (A–Q) Jejunal mucosa mRNA expression of *CCNB1* (A), *CDK1* (B), *DLL1* (C), *OLFM4* (D), *BMI* (E), *LGR5* (F), *BECLIN1* (G), *ERN1* (H), *P53* (I), *SLC2A2* (J), *SLC5A1* (K), *CALB1* (L), *TNFα* (M), *FASL* (N), *Caspase-3* (O), *PDGFR* (P), and *BCL2* (Q), *n* = 6. Data were shown as the mean ± SEM, data with different superscript letters (a, b, and c) were significantly different (*p* < 0.05).

**Figure S4.** **Jejunal mucosa protein expression of proliferation, autophagy, differentiation, and apoptotic genes.** (A) Jejunal mucosa protein expressions of CDK1, LGR5, SLC2A2, SLC5A1, and CALB1 are shown as fold change during FIM. (B–F) The relative protein expression levels of CDK1 (B), LGR5 (C), SLC2A2 (D), SLC5A1 (E), and CALB1 (F) are shown as fold change during FIM. (G) Jejunal mucosa protein expression of BCL2, BECLIN1, and LC3B. (H–J) The relative protein expression levels of BCL2 (H), BECLIN1 (I), and LC3B (J) during FIM. *n* = 3. Data were shown as the mean ± SEM, data with different superscript letters (a, b, and c) were significantly different (*p* < 0.05).

**Figure S5. Immunofluorescence staining.** (A) Ki67; (B) SLC2A2; (C) SLC5A1; (D) CALB1. Scale bars: 100 μm, *n* = 3.

**Figure S6. Analysis of the jejunal transcriptome data during FIM.** (A) Graphs of scale independence, mean connectivity, and scale-free topology, the appropriate soft-power was 8. (B) Cluster dendrogram of the co-expression network modules (1-TOM). (C–E) Blue (C), greenyellow (D), and yellow (E) module scatter plot related to crypt depth. (F, G) red (F) and blue (G) module scatter plot related to villus/crypt ratio. Key genes were screened out in the upper-right area where |GS| > 0.5 and MM > 0.7 (TOM, topological overlap matrix. GS, gene signiﬁcance. MM, module membership). (H–S) qRT-PCR of gene expression in the TGF-beta signaling pathway includes *BMP5* (H), *MYC* (I), *TGFβ2* (J), *SMAD9* (K), *BMP4* (L), *TGIF2* (M), *TGFBR2* (N), *NBL1* (O), *RGMB* (P), *TGFβ3* (Q), *RBL1* (R), and *FST* (S). Data were shown as the mean ± SEM, Data with different superscript letters (a, b, and c) were significantly different (*p* < 0.05), *n* = 6.

**Figure S7. Series test of cluster analysis of differentially expressed genes in the liver transcriptome.** *n* = 3.

**Figure S8.** **Detection of liver function and the expression of bile acid synthesis-related genes.** (A, B) Detection of liver (A) and serum (B) bile acid concentrations, *n* = 3. (C–F) qRT-PCR of gene expression in bile acid synthesis pathways includes *CYP7A1* (C), *CYP46A1* (D), *CYP7B1* (E), and *CYP27A1* (F), *n* = 6. Data were shown as the mean ± SEM, data with different superscript letters (a, b, and c) were significantly different (*p* < 0.05).

**Figure S9.** **Metabolite Heatmaps.** (A) Metabolite heatmap based on commonly enriched pathways between cecal metabolites and jejunal transcriptome. (B) Metabolite heatmap based on commonly enriched pathways between cecal metabolites and liver transcriptome. *n* = 6.

**Figure S10.** **The timeline of FIM.**
